# No major role of norepinephrine transporter gene variations in the cardiostimulant effects of MDMA

**DOI:** 10.1007/s00228-017-2392-2

**Published:** 2017-12-02

**Authors:** Patrick Vizeli, Henriette E. Meyer zu Schwabedissen, Matthias E. Liechti

**Affiliations:** 1 Division of Clinical Pharmacology and Toxicology, Department of Biomedicine, Department of Clinical Research, University Hospital Basel, University of Basel, Schanzenstrasse 55, 4056 Basel, Switzerland; 2 Biopharmacy, Department of Pharmaceutical Sciences, University of Basel, Basel, Switzerland

**Keywords:** MDMA, Norepinephrine transporter, SLC6A2, Pharmacogenetics, Cardiostimulation

## Abstract

**Purpose:**

Methylenedioxymethamphetamine (MDMA, ecstasy) is used recreationally and frequently leads to sympathomimetic toxicity. MDMA produces cardiovascular and subjective stimulant effects that were shown to partially depend on the norepinephrine transporter (NET)-mediated release of norepinephrine and stimulation of α_1_-adrenergic receptors. Genetic variants, such as single-nucleotide polymorphisms (SNPs), of the NET gene (*SLC6A2*) may explain interindividual differences in the acute stimulant-type responses to MDMA in humans.

**Methods:**

We characterized the effects of common genetic variants of the *SLC6A2* gene (rs168924, rs47958, rs1861647, rs2242446, and rs36029) on cardiovascular and subjective stimulation after MDMA administration in 124 healthy subjects in a pooled analysis of eight double-blind, placebo-controlled studies.

**Results:**

Carriers of the GG genotype of the *SLC6A2* rs1861647 SNP presented higher elevations of heart rate and rate-pressure product after MDMA than subjects with one or no G alleles. Subjects with a C allele in the *SLC6A2* rs2242446 SNP presented higher elevations of the heart rate after MDMA administration compared with the TT genotype. Subjects with the AA genotype of the *SLC6A2* rs36029 SNP presented higher elevations of mean arterial pressure and rate pressure product after MDMA administration than carriers of the G allele. The *SLC6A2* rs168924 and rs47958 SNPs did not alter the response to MDMA.

**Conclusions:**

Genetic polymorphisms of the SLC6A2 gene weakly moderated the acute cardiovascular response to MDMA in controlled studies and may play a minor role in adverse cardiovascular events when MDMA is used recreationally.

**Electronic supplementary material:**

The online version of this article (10.1007/s00228-017-2392-2) contains supplementary material, which is available to authorized users.

## Introduction

3,4-Methylenedioxymethamphetamine (MDMA; ecstasy) is used recreationally for its ability to enhance empathic feelings and sociability [1, 2]. MDMA has also been investigated as a treatment for posttraumatic stress disorder [3, 4]. However, MDMA produces adverse effects, including cardio- and psychostimulant effects to varying degrees [5–7]. The sympathomimetic effects of MDMA vary across subjects, and high blood pressure responses were observed in a few subjects [6, 8]. The response to MDMA varies between subjects, and genetic variations may explain some of this interindividual variation [9–11]. For example, genetic variations of the enzymes that are involved in MDMA metabolism (mainly CYP2D6) have been shown to affect plasma levels of MDMA and its metabolites [9, 10, 12] and moderate the pharmacokinetics and partially the pharmacodynamics of MDMA. However, genetic variants of the pharmacological targets of MDMA [7, 13] may also moderate its pharmacodynamic effects, but such effects have not yet been studied.

MDMA interacts with presynaptic monoamine transporters and mainly causes the transporter-mediated efflux of serotonin (5-hydroxytryptamine [5-HT]) and norepinephrine (NE; [14–17]). The transporter-mediated efflux of NE has been suggested to critically contribute to the cardio- and psychostimulant effects of MDMA [7, 13, 18, 19]. The solute carrier family 6 (neurotransmitter transporter, NE transporter [NET]), member 2 (*SLC6A2*) is a crucial player in the noradrenergic system and involved in the mechanism of action of MDMA in humans. Inhibition of the NET significantly attenuated the sympathomimetic stimulant-like effects of MDMA and other stimulant-type substances [7, 20]. In a controlled study in healthy subjects, the NET inhibitor reboxetine reduced the MDMA-induced elevations of blood pressure and heart rate and subjective stimulation and increased the pupil diameter at rest and after light [7, 21], indicating a role for the NET in mediating the MDMA response [22]. Similarly, pretreatment with the NET inhibitor atomoxetine attenuated d-amphetamine-induced elevations of blood pressure and self-reported ratings of feeling “stimulated” [20]. Another study in humans showed a similar reduction of cardiostimulant responses to cocaine after treatment with the NET inhibitor atomoxetine [23].

Several genetic variations of the *SLC6A2* gene that are caused by single-nucleotide polymorphisms (SNPs) are associated with different functional phenotypes. However, the roles of these genotypes in the effects MDMA have not yet been investigated. Therefore, we focused on validated, polymorphic (minor allele frequency in Caucasians > 0.1), and potentially functionally relevant variants of *SLC6A2*. Specifically, the G allele of the *SLC6A2* rs168924 SNP was associated with hypertension in Japanese patients [24] but lower blood pressure in Caucasians [25]. Subjects with the AA genotype of the *SLC6A2* rs1861647 SNP or CC genotype of the *SLC6A2* rs47958 SNP had higher subjective elation scores in response to d-amphetamine compared with carriers of the G allele [26] or A allele [27], respectively. The *SLC6A2* rs2242446 SNP was shown to influence blood pressure during exercise [28]. Additionally, an association was found between the rs2242446 SNP and recurrent depression [29] and antidepressant response to the 5-HT/NE transporter inhibitor milnacipran [30]. Finally, the *SLC6A2* rs36029 SNP was shown to be significantly associated with alcohol dependence [31].

The present study investigated the impact of genotypes within the noradrenergic system on the effects of MDMA. We evaluated whether the *SLC6A2* rs168924, rs47958, rs1861647, rs2242446, and rs36029 SNPs influence the cardiovascular and subjective stimulant effects of MDMA. MDMA-induced peak increases in the rate-pressure product (RPP) and subjective ratings of stimulation were considered the two primary endpoints. Plasma concentrations of MDMA and NE were determined to exclude possible confounding effects on the influence of genotype.

## Methods

### Study design

This was a pooled analysis of eight Phase I double-blind, placebo-controlled, crossover studies in healthy subjects that used similar methods [7, 13, 21, 32–36]. These studies included a total of 136 healthy subjects. Seven studies included 16 subjects each, for a total of 112 subjects, who received 125 mg MDMA twice, once alone, and once after pretreatment with a medication [7, 13, 21, 32–36]. An additional study included 24 subjects who received 125 mg MDMA once alone, placebo, or other treatments [36]. In the present analysis, only data from the MDMA-alone and placebo sessions were used. In all of the studies, the washout periods between single-dose administrations of MDMA were at least 7 days to exclude carry-over effects. The studies were all registered at ClinicalTrials.gov (NCT00886886, NCT00990067, NCT01136278, NCT01270672, NCT01386177, NCT01465685, NCT01771874, and NCT01951508). All of the studies were approved by the local ethics committee and Swiss Agency for Therapeutic Products (Swissmedic). The studies were conducted in accordance with the Declaration of Helsinki. MDMA administration in healthy subjects was authorized by the Swiss Federal Office for Public Health (BAG), Bern, Switzerland. Informed consent was obtained from all of the participants who were included in the studies. All of the subjects were paid for their participation. Pharmacokinetic and safety data from these studies have been reported elsewhere [6, 9, 10]. In all studies, test sessions took place in a quiet hospital research ward with no more than two research subjects present per session. The participants were comfortably lying in hospital beds and were mostly listening to music and did not engage in physical activities. MDMA was given without food in the fasting state in the morning at 8:00–9:00 a.m.. A small standardized lunch was served at 12:00–1:00 p.m.

### Subjects

A total of 136 healthy European/Caucasian subjects, 18–44 years old (mean ± SD = 24.8 ± 4 years), were recruited from the University of Basel campus and participated in the study. One genotyping sample was missing, three participants did not give consent for genotyping, and eight subjects participated twice, and only the first participation was included, resulting in data from 124 subjects. The mean ± SD body weight was 68 ± 10 kg (range 46–90 kg).

The exclusion criteria included a history of psychiatric disorders, physical illness, a lifetime history of using illicit drugs more than five times (with the exception of past cannabis use), illicit drug use within the past 2 months, and illicit drug use during the study, determined by urine tests that were conducted before the test sessions as reported in detail elsewhere [13, 21, 32, 33]. Thirty-eight subjects had prior illicit drug experiences (1–5 times), of which 16 subjects had previously used MDMA (1–2 times), 7 amphetamine or methamphetamine (1 time), 9 cocaine (1–3 times), 6 lysergic acid diethylamide (1 time), and 11 psilocybin (1–4 times).

### Study drug

(±)MDMA hydrochloride (Lipomed AG, Arlesheim, Switzerland) was administered orally in a single dose of 125 mg, prepared as gelatin capsules (Bichsel Laboratories, Interlaken, Switzerland). Similar amounts of MDMA are found in ecstasy pills [37] and have been used in clinical studies in patients [3, 4]. The doses were not adjusted for body weight or sex. The dose per body weight (mean ± SD) was 1.9 ± 0.3 mg/kg (1.7 ± 0.2 mg/kg for men and 2.1 ± 0.3 mg/kg for women, range 1.4–2.7 mg/kg).

### Cardiovascular effects

Blood pressure and heart rate were assessed repeatedly before and 0, 0.33, 0.67, 1, 1.5, 2, 2.5, 3, 4, 5, and 6 h after MDMA or placebo administration. Systolic and diastolic blood pressure and heart rate were measured using an automatic oscillometric device (OMRON Healthcare Europe NA, Hoofddorp, Netherlands). The measurements were performed in duplicate at an interval of 1 min and after a resting time of at least 10 min. The averages were calculated for the analysis. Mean arterial pressure (MAP) was calculated as diastolic blood pressure + (systolic blood pressure − diastolic blood pressure) / 3. The RPP was calculated as *systolic blood pressure* × *heart rate* and was considered the primary cardiovascular measure that reflected overall cardiovascular stimulation.

### Subjective effects

To assess subjective stimulation, a visual analog scale of “stimulated” was presented as a 100-mm horizontal line (0–100%), marked from “not at all” on the left to “extremely” on the right [1]. The scale was administered before and 0, 0.33, 0.67, 1, 1.5, 2, 2.5, 3, 4, 5, and 6 h after MDMA or placebo administration.

### Plasma concentrations of MDMA and norepinephrine

Plasma levels of MDMA were determined before and 0.5, 1, 1.5, 2, 3, 4, and 6 h after drug administration [34]. Plasma levels of NE were measured before and 2 h after drug administration as described previously [7, 38].

### Pupillometry

Pupillometry was performed 1 h before and 0, 0.33, 0.66, 1, 1.5, 2, 2.5, 3, 4, 5, and 6 h after MDMA or placebo administration. Pupil function was measured under standardized dark-light conditions using a hand-held PRL-200 infrared pupillometer (NeurOptics, Irvine, CA) as reported previously in detail [21]. Dark-adapted pupil diameter and minimal pupil diameter after a light stimulus were assessed.

### Genotyping

Genomic DNA was extracted from whole blood using the QIAamp DNA Blood Mini Kit (Qiagen, Hombrechtikon, Switzerland) and automated QIAcube system. Genotyping was performed using commercial TaqMan SNP genotyping assays (LuBio Science, Lucerne, Switzerland) and the TaqMan Genotyping Master Mix. Fluorescence was detected using the ViiA7 real-time PCR system. We assayed the following *SLC6A2* SNPs: rs168924 (assay: C____581568_10), rs1861647 (assay: C___1232469_30), rs47958 (0.39, assay: C___3020083_10), rs2242446 (assay: C__26354911_10), rs36029 (C___1232432_10). We also assayed the following ADRA1A SNP: rs1048101 (assay: C___2696454_30). However, due to inconsistency with the Hardy-Weinberg equilibrium, we excluded the ADRA1A rs1048101 SNP from further analysis. The rs1861647 genotype could not be determined in one subject.

### Statistical analysis

The statistical analyses were performed using Statistica 12 software (StatSoft, Tulsa, OK, USA). For repeatedly measured data, peak effects (E_max_) and areas under the effect-time curve (AUEC) from 0 to 6 h values were determined for MDMA and placebo. Differences in E_max_ and AUEC values (MDMA-placebo) were then analyzed using one-way analysis of variance (ANOVA), with genotype as the between-group factor, followed by the Tukey post hoc test. The primary analysis did not control for the multiple comparisons, but a secondary analysis was conducted using Bonferroni correction for the five SNPs. To account for differences in plasma concentrations of MDMA that were caused by differences in body weight, dosing, or metabolizing enzymes [9, 10], the area under the MDMA plasma concentration-time curve from 0 to 6 h (AUC) was included as a covariate in the ANOVAs, and we report the corrected statistics. Additionally, moderating effects of sex were explored by adding sex as a between-subjects factor in the ANOVAs. E_max_ values were obtained directly from the observed data, and AUC and AUEC curves were calculated using the linear-log trapezoidal method in Phoenix WinNonlin 6.4 (Certara, Princeton, NJ).

## Results

Effects of the SNPs on the maximum response (E_max_) to MDMA are shown in Table 1. Supplementary Table S1 shows the data without adjustment for MDMA plasma concentrations. Supplementary Table S2 shows effects of the SNPs on the overall response to MDMA (AUEC values).

**Table 1:** Effects of the SLC6A2 SNPs rs168924, rs47958, rs1861647, rs2242446, and rs36029 on the maximum response to MDMA (mean ± SD and statistics)

**SNP rs168924**	Number of genotypes	AA	AG	GG	F	*p* value	*p* value (Bonferroni corr.)
*N* (%)		95 (77)	26 (21)	3 (2)			
Female, *N* (%)		52 (55)	12 (46)	0 (0)			
MDMA plasma concentration AUC (ng/ml)	N 95, 26, 3	956 ± 201	957 ± 226	784 ± 126	1.03	NS	NS
Norepinephrine Δplasma concentration at 2 h (pg/ml)	N 68, 14, 2	0.5 ± 0.6	0.8 ± 0.7	0.5 ± 1.0	0.73	NS	NS
Subjective stimulation, ΔE_max_ (%)	N 95, 26, 3	63 ± 33	72 ± 33	41 ± 42	1.07	NS	NS
Mean arterial pressure, ΔE_max_, (mmHg)	N 95, 26, 3	18 ± 10	20 ± 9	13 ± 15	0.66	NS	NS
Heart rate, ΔE_max_ (bpm)	N 95, 26, 3	19 ± 15	18 ± 15	17 ± 17	0.04	NS	NS
Rate pressure product, ΔE_max_ (mmHg/min)	N 95, 26, 3	4678 ± 2976	4869 ± 3093	3937 ± 3316	0.05	NS	NS
Pupil size, ΔE_max_ (mm)	N 93, 25, 3	0.9 ± 0.5	1.0 ± 0.4	0.7 ± 0.4	0.62	NS	NS
Pupil size after light, ΔE_max_ (mm)	N 93, 25, 3	2.0 ± 0.7	2.1 ± 0.6	1.5 ± 0.6	1.04	NS	NS
**SNP rs47958**	Number of genotypes	AA	AC	CC	F	*p* value	*p* value (Bonferroni corr.)
*N* (%)		27 (22)	58 (47)	39 (31)			
Female, *N* (%)		13 (48)	29 (50)	22 (56)			
MDMA plasma concentration AUC (ng/ml)	N 27, 58, 39	941 ± 177	954 ± 234	957 ± 181	0.06	NS	NS
Norepinephrine Δplasma concentration at 2 h (pg/ml)	N 18, 41, 25	0.4 ± 0.8	0.5 ± 0.7	0.6 ± 0.6	0.29	NS	NS
Subjective stimulation, ΔE_max_ (%)	N 27, 58, 39	62 ± 34	64 ± 34	67 ± 32	0.13	NS	NS
Mean arterial pressure, ΔE_max_ (mmHg)	N 27, 58, 39	21 ± 10	17 ± 8	18 ± 11	1.99	NS	NS
Heart rate, ΔE_max_ (bpm)	N 27, 58, 39	21 ± 14	20 ± 16	14 ± 14	2.41	NS	NS
Rate pressure product, ΔE_max_ (mmHg/min)	N 27, 58, 39	5391 ± 2654	4882 ± 3127	3950 ± 2898	2.36	NS	NS
Pupil size, ΔE_max_ (mm)	N 27, 57, 37	0.8 ± 0.3	1.0 ± 0.4	0.9 ± 0.6	1.48	NS	NS
Pupil size after light, ΔE_max_ (mm)	N 27, 57, 37	1.9 ± 0.5	2.1 ± 0.6	1.9 ± 1.0	0.72	NS	NS
**SNP rs1861647**	Number of genotypes	AA	AG	GG	F	*p* value	*p* value (Bonferroni corr.)
*N* (%)		12 (10)	55 (45)	56 (46)			
Female, *N* (%)		6 (50)	28 (51)	29 (52)			
MDMA plasma concentration AUC (ng/ml)	N 12, 55, 56	917 ± 172	943 ± 213	966 ± 205	0.36	NS	NS
Norepinephrine Δplasma concentration at 2 h (pg/ml)	N 6, 39, 38	0.3 ± 0.6	0.6 ± 0.6	0.6 ± 0.8	0.52	NS	NS
Subjective stimulation, ΔE_max_ (%)	N 12, 55, 56	73 ± 29	62 ± 32	64 ± 36	0.79	NS	NS
Mean arterial pressure, ΔE_max_ (mmHg)	N 12, 55, 56	17 ± 11	17 ± 9	19 ± 10	0.42	NS	NS
Heart rate, ΔE_max_ (bpm)	N 12, 55, 56	13 ± 11	15 ± 14**	23 ± 15	4.90	0.009	0.045
Rate pressure product, ΔE_max_ (mmHg/min)	N 12, 55, 56	4025 ± 2235	4018 ± 3099*	5527 ± 2851	3.74	0.027	NS
Pupil size, ΔE_max_ (mm)	N 12, 53, 55	0.8 ± 0.3	0.9 ± 0.5	0.9 ± 0.4	0.37	NS	NS
Pupil size after light, ΔE_max_ (mm)	N 12, 53, 55	2.1 ± 0.6	1.9 ± 0.8	2.1 ± 0.6	0.94	NS	NS
**SNP rs2242446**	Number of genotypes	CC	CT	TT	F	*p* value	*p* value (Bonferroni corr.)
*N* (%)		15 (12)	50 (40)	59 (48)			
Female, *N* (%)		6 (40)	26 (52)	32 (54)			
MDMA plasma concentration AUC (ng/ml)	N 15, 50, 59	956 ± 174	942 ± 217	960 ± 205	0.09	NS	NS
Norepinephrine Δplasma concentration at 2 h (pg/ml)	N 10, 36, 38	0.3 ± 0.8	0.6 ± 0.7	0.6 ± 0.6	0.55	NS	NS
Subjective stimulation, ΔE_max_ (%)	N 15, 50, 59	72 ± 33	65 ± 31	61 ± 36	0.77	NS	NS
Mean arterial pressure, ΔE_max_ (mmHg)	N 15, 50, 59	24 ± 11	17 ± 8***	17 ± 10***	3.61	0.030	NS
Heart rate, ΔE_max_ (bpm)	N 15, 50, 59	26 ± 18	21 ± 16	14 ± 12***	5.08	0.008	0.038
Rate pressure product, ΔE_max_ (mmHg/min)	N 15, 50, 59	6205 ± 2997	5042 ± 3191	4027 ± 2642***	4.35	0.015	NS
Pupil size, ΔE_max_ (mm)	N 15, 48, 58	0.7 ± 0.2	0.9 ± 0.4	0.9 ± 0.5	1.81	NS	NS
Pupil size after light, ΔE_max_ (mm)	N 15, 48, 58	1.9 ± 0.5	1.9 ± 0.5	2.1 ± 0.9	0.97	NS	NS
**SNP rs36029**	Number of genotypes	AA	AG	GG	F	*p* value	*p* value (Bonferroni corr.)
*N* (%)		46 (37)	56 (45)	22 (18)			
Female, *N* (%)		19 (41)	32 (57)	13 (59)			
MDMA plasma concentration AUC (ng/ml)	N 46, 56, 22	911 ± 180	972 ± 212	989 ± 230	1.58	NS	NS
Norepinephrine Δplasma concentration at 2 h (pg/ml)	N 33, 37, 14	0.7 ± 0.7	0.4 ± 0.7	0.5 ± 0.5	2.57	NS	NS
Subjective stimulation, ΔE_max_ (%)	N 46, 56, 22	65 ± 33	64 ± 33	62 ± 38	0.50	NS	NS
Mean arterial pressure, ΔE_max_ (mmHg)	N 46, 56, 22	20 ± 10	16 ± 10	17 ± 9	4.82	0.010	0.049
Heart rate, ΔE_max_ (bpm)	N 46, 56, 22	21 ± 16	17 ± 15	16 ± 14	2.12	NS	NS
Rate pressure product, ΔE_max_ (mmHg/min)	N 46, 56, 22	5419 ± 2974	4317 ± 3067	4169 ± 2608	3.47	0.034	NS
Pupil size, ΔE_max_ (mm)	N 46, 54, 21	0.9 ± 0.3	0.9 ± 0.4	0.8 ± 0.6	0.62	NS	NS
Pupil size after light, ΔE_max_ (mm)	N 46, 54, 21	1.9 ± 0.5	2.1 ± 0.7	1.9 ± 1.0	1.25	NS	NS

F and *p* values are from ANCOVAs (except for the MDMA concentrations) with MDMA AUC as covariate to account for differences in MDMA concentrations

*N* number of subjects, *SNP* single nucleotide polymorphism, *E*_*max*_ peak effect, *AUC* area under the concentration-time curve from 0 to 6 h, *NS* not significant, *Δ* values are change scores from placebo (mdma-placebo)

*p < 0.05; **p < 0.01 compared to rs1861647 GG; ***p < 0.05 compared to rs2242446 CC

### Genotyping

The distribution of the alleles and genotypes did not differ from the distributions that were reported elsewhere in Caucasian cohorts (Ensembl database release 88, Mar 2017). The minor allele frequencies for rs168924, rs47958, rs1861647, rs2242446, rs36029, and rs1048101 were G (29 [13%]), A (112 [45%]), A (79 [32%]), C (80 [33%]), G (100 [40%]), and G (107 [43%]), respectively. The tested genetic variants were consistent with the Hardy-Weinberg equilibrium (*p* > 0.05) with the exception of rs1048101 (*p* = 0.01).

### Plasma concentrations of MDMA and norepinephrine

Plasma concentrations of MDMA and norepinephrine did not differ between the different genotype groups (Table 1 and Supplementary Table S1).

### Subjective effects

None of the examined polymorphisms influenced subjective stimulation that was induced by MDMA (Table 1, Supplementary Tables S1 and S2).

### Pupillary effects

None of the examined polymorphisms influenced the MDMA-induced change in pupillary size before and after light stimulus (Table 1 and Supplementary Tables S1 and S2).

### Cardiovascular effects

**Fig. 1:**
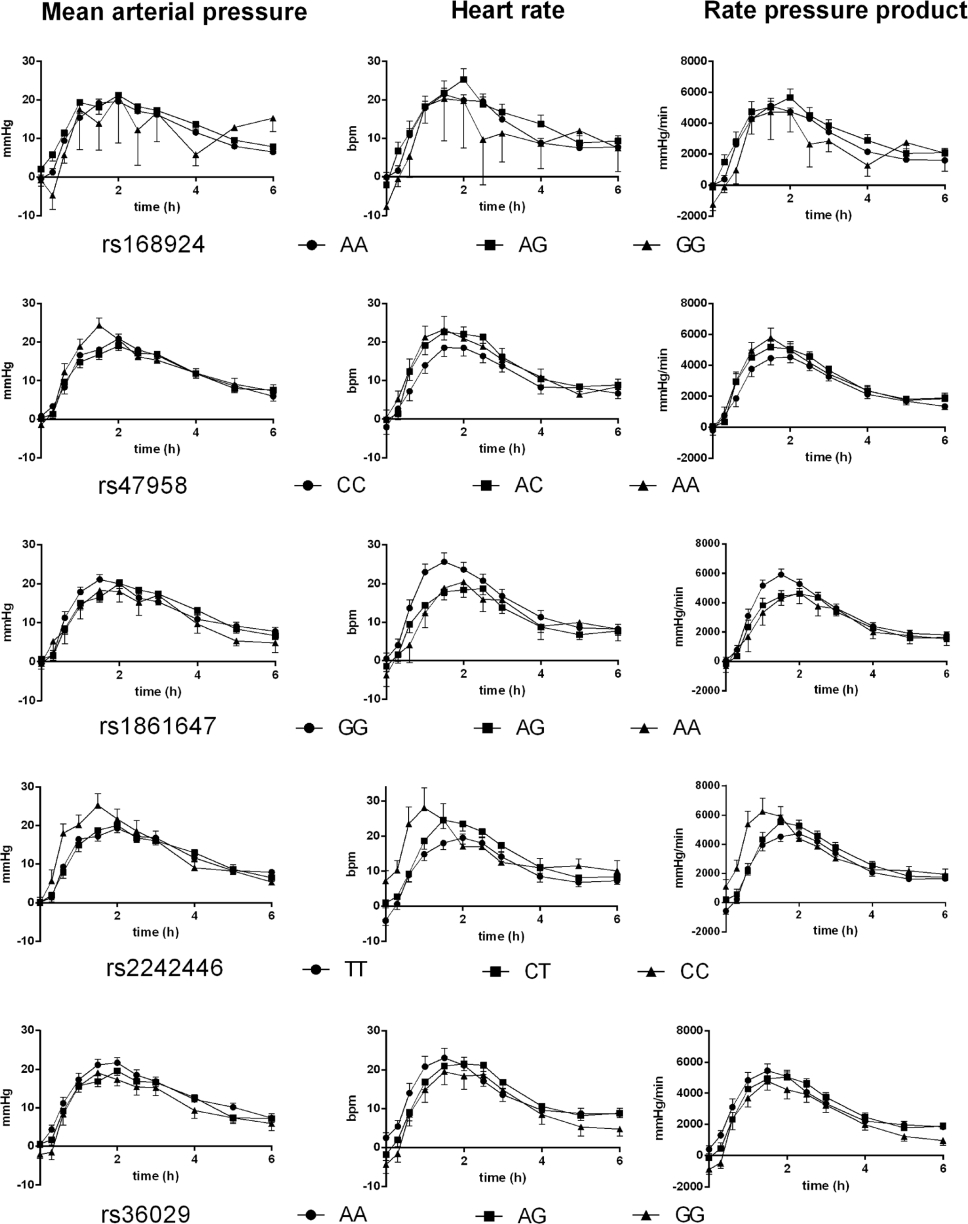
Effects of common genetic variants of the *SLC6A2* gene (rs168924, rs47958, rs1861647, rs2242446, and rs36029) on cardiovascular stimulation after MDMA administration in 124 healthy subjects. Homozygous carriers of *SLC6A2* rs1861647 G allele presented higher elevations of heart rate and rate-pressure product after MDMA than subjects with one G allele. Subjects with the CC genotype of the *SLC6A2* rs2242446 SNP presented higher elevations of heart rate, mean arterial pressure, and rate-pressure product after MDMA administration compared with the TT genotype group. The *SLC6A2* rs168924 and rs47958 SNPs did not significantly alter the response to MDMA. The corresponding maximal effects and statistics are shown in Table 1. The data are expressed as mean ± SEM. MDMA or placebo was administered at time = 0

The effects of the polymorphisms on elevations of MAP, heart rate, and RPP in response to MDMA (adjusted for differences in plasma MDMA concentrations) are shown in Table 1 and Fig. 1. The rs1861647 SNP located in *SLC6A2* significantly altered the elevations of heart rate and RPP after MDMA administration. The effect on the heart rate remained significant after Bonferroni correction for multiple testing (*p* < 0.05). Subjects with the GG genotype had significantly higher elevations of heart rate and RPP after MDMA administration than subjects with the AG genotype. When we combined the AA and AG genotype groups, subjects with the GG genotype presented higher elevations of heart rate and RPP than carriers of the minor A allele (*F*_1,120_ = 9.79, *p* < 0.01 and *F*_1,120_ = 7.53, *p* < 0.01, respectively). These effects remained significant after Bonferroni correction for multiple testing (*p* < 0.02 and *p* < 0.04, respectively).

The rs2242446 SNP significantly moderated the elevation of RPP after MDMA administration, which was attributable to significant moderating effects on MDMA-induced changes in MAP and heart rate. The CC genotype presented elevations of heart rate, MAP, and RPP after MDMA administration compared with the TT genotype. When we combined the rs2242446 CT and CC genotype groups, subjects with the TT genotype presented lower elevations of heart rate and RPP than carriers of the C allele (*F*_1,121_ = 7.81, *p* < 0.01 and *F*_1,121_ = 5.64, *p* < 0.05, respectively). The difference between the genotype groups and the combined groups (TT and CT/CC) in the effects of MDMA on heart rate remained significant after Bonferroni correction for multiple testing (*p* < 0.04 and *p* < 0.04, respectively).

Significant main effects of the rs36029 SNP on MDMA-induced elevations of MAP and RPP were found. When we combined the rs36029 AG and GG genotype groups, subjects with the AA genotype presented higher elevations of MAP, heart rate, and RPP than carriers of the G allele (*F*_1,121_ = 9.870, *p* < 0.01; *F*_1,121_ = 4.24, *p* < 0.05; and *F*_1,121_ = 6.91, *p* < 0.01, respectively). The difference in the effects of MDMA on MAP between the genotype groups and difference in the effects of MDMA on MAP and RPP between the combined groups (AA and AG/GG) remained significant after Bonferroni correction for multiple testing (*p* < 0.05, *p* < 0.02, and *p* < 0.05, respectively).

The effects of the rs1861647, rs2242446, and rs36029 SNPs on the peak response to MDMA were similar when the analyses were performed without using MDMA plasma concentrations as covariate in the ANOVAs (Supplementary Table S1). However, none of the SNPs altered the overall cardiovascular response to MDMA as expressed by the AUEC values (Supplementary Table S2) with the exception of the effect of the rs36029 SNP on the MAP.

The rs168924 and rs47958 SNPs did not alter the effects of MDMA. When we applied Bonferroni correction for multiple testing (for five SNPs), none of the statistical findings remained significant in the additive genotype group models (Table 1) with the exception of the effect of the rs2242446 SNP on heart rate, and sex did not alter the influence of the SNPs on the response to MDMA (Supplementary Table S3).

## Discussion

The present study investigated the effect of interindividual differences in the *SLC6A2* gene on the cardiovascular and subjective stimulant response to MDMA. None of the investigated SNPs moderated the subjective stimulant effects of MDMA. Three SNPs of the *SLC6A2* gene (rs1861647, rs2242446, and rs36029) influenced the cardiovascular response to MDMA. However, the effect sizes for these genetic variants were rather small and not very robust. In fact, Bonferroni correction of the data for the five SNPs resulted in the loss of most statistical significance. Thus, although the NET has been implicated in the stimulant-type response to MDMA [7], the genetic variants of the NET gene (*SLC6A2*) that were evaluated herein only minimally influenced the response to MDMA.

To our knowledge, the present study was the first to explore the role of SNPs of the *SLC6A2* gene in the response to MDMA. Thus, no comparisons can be made with other studies that tested MDMA. In a previous study, C-allele carriers of the *SLC6A2* rs2242446 SNP presented higher blood pressure after physical exercise [28], which is consistent with the greater blood pressure response in the present study following the administration of a pharmacological stimulant. Additionally, plasma NE concentrations after exercise differed between different rs2242446 genotypes [28]. In the present study, however, no differences in NE levels after MDMA administration were found between genotype groups. The effects of the *SLC6A2* rs1861647 and rs47958 SNPs on the response to d-amphetamine have previously been reported [26, 27, 39]. Initial studies showed that subjects with the AA genotype of rs1861647 had higher vigor scores after d-amphetamine administration [26]. Additionally, subjects with the CC genotype of rs47958 had higher positive mood scores [27]. However, a subsequent larger replication study found no influence of the different rs1861647 and rs47958 genotypes on the response to d-amphetamine [39] as similarly documented in the present study for the subjective response to MDMA. However, the effects of *SLC6A2* SNPs on the cardiovascular response to d-amphetamine or MDMA have not been studied previously; therefore, the role of the rs1861647 SNP in the cardiovascular stimulant effects of MDMA that was identified in the present study needs further investigation.

Additionally, none of the NET genotypes moderated the MDMA-induced increase in pupil size [21].

The present study has several limitations. First, the sample size was relatively small when considering the mostly small effect sizes for the influence of genetic variants on the MDMA response. Additionally, significant findings in the additive genotype models were mostly lost after Bonferroni correction. Confirmation in studies with larger samples is needed. However, we unlikely missed very large effect sizes for the influence of these genetic variants or possible haplotypes. Second, the study was conducted in mostly young and healthy volunteers. Therefore, the findings cannot necessarily be generalized to people with hypertension or other cardiovascular risk factors. Third, SNPs of the genes of other targets of MDMA, such as the 5-HT transporter [13, 18], may also be involved but were not tested in the present study. However, we considered the moderating effects of known genetic variants that influence the metabolism of MDMA [9, 10] by accounting for interindividual differences in plasma MDMA concentrations.

In conclusion, the present study investigated the influence of genetic polymorphisms of the *SLC6A2* gene on the response to MDMA. Three SNPs of the *SLC6A2* gene (rs2242446, rs1861647, and rs36029) weakly altered the cardiovascular effects of MDMA in healthy subjects. It can be assumed that these genetic polymorphisms may play a minor role in adverse cardiovascular events when MDMA is used recreationally or therapeutically.

## Electronic supplementary material

ESM 1 (XLSX 19 kb)

ESM 2 (XLSX 16 kb)

ESM 3 (XLSX 17 kb)

## References

[1] Hysek CM, Schmid Y, Simmler LD, Domes G, Heinrichs M, Eisenegger C, Preller KH, Quednow BB, Liechti ME (2014). MDMA enhances emotional empathy and prosocial behavior. Soc Cogn Affect Neurosci.

[2] Wardle MC, Kirkpatrick MG, de Wit H (2014). ‘Ecstasy’ as a social drug: MDMA preferentially affects responses to emotional stimuli with social content. Soc Cogn Affect Neurosci.

[3] Oehen P, Traber R, Widmer V, Schnyder U (2013). A randomized, controlled pilot study of MDMA (±3,4-methylenedioxymethamphetamine)-assisted psychotherapy for treatment of resistant, chronic post-traumatic stress disorder (PTSD). J Psychopharmacol.

[4] Mithoefer MC, Wagner MT, Mithoefer AT, Jerome I, Doblin R (2010). The safety and efficacy of ±3,4-methylenedioxymethamphetamine-assisted psychotherapy in subjects with chronic, treatment-resistant posttraumatic stress disorder: the first randomized controlled pilot study. J Psychopharmacol.

[5] Ramaekers JG, Kuypers KP, Samyn N (2006). Stimulant effects of 3,4-methylenedioxymethamphetamine (MDMA) 75 mg and methylphenidate 20 mg on actual driving during intoxication and withdrawal. Addiction.

[6] Vizeli P, Liechti ME (2017). Safety pharmacology of acute MDMA administration in healthy subjects. J Psychopharmacol.

[7] Hysek CM, Simmler LD, Ineichen M, Grouzmann E, Hoener MC, Brenneisen R, Huwyler J, Liechti ME (2011). The norepinephrine transporter inhibitor reboxetine reduces stimulant effects of MDMA (“ecstasy”) in humans. Clin Pharmacol Ther.

[8] Liechti ME, Gamma A, Vollenweider FX (2001). Gender differences in the subjective effects of MDMA. Psychopharmacology.

[9] Vizeli P, Schmid Y, Prestin K, Meyer zu Schwabedissen HE, Liechti ME (2017). Pharmacogenetics of ecstasy: CYP1A2, CYP2C19, and CYP2B6 polymorphisms moderate pharmacokinetics of MDMA in healthy subjects. Eur Neuropsychopharmacol.

[10] Schmid Y, Vizeli P, Hysek CM, Prestin K, Meyer zu Schwabedissen HE, Liechti ME (2016). CYP2D6 function moderates the pharmacokinetics and pharmacodynamics of MDMA in a controlled study in healthy subjects. Pharmacogenet Genomics.

[11] Bershad AK, Weafer JJ, Kirkpatrick MG, Wardle MC, Miller MA, de Wit H (2016). Oxytocin receptor gene variation predicts subjective responses to MDMA. Soc Neurosci.

[12] de la Torre R, Yubero-Lahoz S, Pardo-Lozano R, Farre M (2012). MDMA, methamphetamine, and CYP2D6 pharmacogenetics: what is clinically relvant?. Front Genet.

[13] Hysek CM, Simmler LD, Nicola V, Vischer N, Donzelli M, Krähenbühl S, Grouzmann E, Hoener MC, Liechti ME (2012). Duloxetine inhibits effects of MDMA (“ecstasy”) in vitro and in humans in a randomized placebo-controlled laboratory study. PLoS One.

[14] Rothman RB, Baumann MH, Dersch CM, Romero DV, Rice KC, Carroll FI, Partilla JS (2001). Amphetamine-type central nervous system stimulants release norepinephrine more potently than they release dopamine and serotonin. Synapse.

[15] Rudnick G, Wall SC (1992). The molecular mechanism of “ecstasy” [3,4-methylenedioxy-methamphetamine (MDMA)]: serotonin transporters are targets for MDMA-induced serotonin release. Proc Natl Acad Sci U S A.

[16] Simmler L, Buser T, Donzelli M, Schramm Y, Dieu LH, Huwyler J, Chaboz S, Hoener M, Liechti ME (2013). Pharmacological characterization of designer cathinones in vitro. Br J Pharmacol.

[17] Verrico CD, Miller GM, Madras BK (2007). MDMA (ecstasy) and human dopamine, norepinephrine, and serotonin transporters: implications for MDMA-induced neurotoxicity and treatment. Psychopharmacology.

[18] Liechti ME, Vollenweider FX (2000). The serotonin uptake inhibitor citalopram reduces acute cardiovascular and vegetative effects of 3,4-methylenedioxymethamphetamine (‘ecstasy’) in healthy volunteers. J Psychopharmacol.

[19] Hysek CM, Fink AE, Simmler LD, Donzelli M, Grouzmann E, Liechti ME (2013). Alpha-adrenergic receptors contribute to the acute effects of MDMA in humans. J Clin Psychopharmacol.

[20] Sofuoglu M, Poling J, Hill K, Kosten T (2009). Atomoxetine attenuates dextroamphetamine effects in humans. Am J Drug Alcohol Abuse.

[21] Hysek CM, Liechti ME (2012). Effects of MDMA alone and after pretreatement with reboxetine, duloxetine, clonidine, carvedilol, and doxazosin on pupillary light reflex. Psychopharmacology.

[22] Newton TF (2011). A perhaps unexpected role of norepinephrine in actions of MDMA. Clin Pharmacol Ther.

[23] Stoops WW, Blackburn JW, Hudson DA, Hays LR, Rush CR (2008). Safety, tolerability and subject-rated effects of acute intranasal cocaine administration during atomoxetine maintenance. Drug Alcohol Depend.

[24] Ono K, Iwanaga Y, Mannami T, Kokubo Y, Tomoike H, Komamura K, Shioji K, Yasui N, Tago N, Iwai N (2003). Epidemiological evidence of an association between SLC6A2 gene polymorphism and hypertension. Hypertens Res.

[25] Zolk O, Ott C, Fromm MF, Schmieder RE (2012). Effect of the rs168924 single-nucleotide polymorphism in the SLC6A2 catecholamine transporter gene on blood pressure in Caucasians. J Clin Hypertens (Greenwich).

[26] Dlugos AM, Hamidovic A, Palmer AA, de Wit H (2009). Further evidence of association between amphetamine response and SLC6A2 gene variants. Psychopharmacology.

[27] Dlugos A, Freitag C, Hohoff C, McDonald J, Cook EH, Deckert J, de Wit H (2007). Norepinephrine transporter gene variation modulates acute response to D-amphetamine. Biol Psychiatry.

[28] Kohli U, Hahn MK, English BA, Sofowora GG, Muszkat M, Li C, Blakely RD, Stein CM, Kurnik D (2011). Genetic variation in the presynaptic norepinephrine transporter is associated with blood pressure responses to exercise in healthy humans. Pharmacogenet Genomics.

[29] Hahn MK, Blackford JU, Haman K, Mazei-Robison M, English BA, Prasad HC, Steele A, Hazelwood L, Fentress HM, Myers R, Blakely RD, Sanders-Bush E, Shelton R (2008). Multivariate permutation analysis associates multiple polymorphisms with subphenotypes of major depression. Genes Brain Behav.

[30] Yoshida K, Takahashi H, Higuchi H, Kamata M, Ito K, Sato K, Naito S, Shimizu T, Itoh K, Inoue K, Suzuki T, Nemeroff CB (2004). Prediction of antidepressant response to milnacipran by norepinephrine transporter gene polymorphisms. Am J Psychiatry.

[31] Clarke TK, Dempster E, Docherty SJ, Desrivieres S, Lourdsamy A, Wodarz N, Ridinger M, Maier W, Rietschel M, Schumann G (2012). Multiple polymorphisms in genes of the adrenergic stress system confer vulnerability to alcohol abuse. Addict Biol.

[32] Hysek CM, Brugger R, Simmler LD, Bruggisser M, Donzelli M, Grouzmann E, Hoener MC, Liechti ME (2012). Effects of the alpha2-adrenergic agonist clonidine on the pharmacodynamics and pharmacokinetics of 3,4-methylenedioxymethamphetamine in healthy volunteers. J Pharmacol Exp Ther.

[33] Hysek CM, Schmid Y, Rickli A, Simmler LD, Donzelli M, Grouzmann E, Liechti ME (2012). Carvedilol inhibits the cardiostimulant and thermogenic effects of MDMA in humans. Br J Pharmacol.

[34] Hysek CM, Simmler LD, Schillinger N, Meyer N, Schmid Y, Donzelli M, Grouzmann E, Liechti ME (2014). Pharmacokinetic and pharmacodynamic effects of methylphenidate and MDMA administered alone and in combination. Int J Neuropsychopharmacol.

[35] Schmid Y, Rickli A, Schaffner A, Duthaler U, Grouzmann E, Hysek CM, Liechti ME (2015). Interactions between bupropion and 3,4-methylenedioxymethamphetamine in healthy subjects. J Pharmacol Exp Ther.

[36] Dolder PC, Muller F, Schmid Y, Borgwardt SJ, Liechti ME (2017) Direct comparison of the acute subjective, emotional, autonomic, and endocrine effects of MDMA, methylphenidate, and modafinil in healthy subjects. Psychopharmacology (in press). 10.1007/s00213-017-4650-5 10.1007/s00213-017-4650-5 PMC5813072 28551715

[37] Brunt TM, Koeter MW, Niesink RJ, van den Brink W (2012). Linking the pharmacological content of ecstasy tablets to the subjective experiences of drug users. Psychopharmacology.

[38] Dunand M, Gubian D, Stauffer M, Abid KA, Grouzmann E (2013). High throughput and sensitive quantitation of plasma catecholamines by ultraperformance liquid chromatography-tandem mass spectrometry using a solid phase microwell extraction plate. Anal Chem.

[39] Hart AB, de Wit H, Palmer AA (2013). Candidate gene studies of a promising intermediate phenotype: failure to replicate. Neuropsychopharmacology.

